# The Fernandes Figueira Institute: a century devoted to mothers, children, and adolescents

**DOI:** 10.1590/S0104-59702024000100001en

**Published:** 2024-04-05

**Authors:** Luiz Antonio Teixeira, Larissa Velasquez de Souza

**Affiliations:** i Universidade Estácio de Sá; Instituto Nacional de Saúde da Mulher, da Criança e do Adolescente Fernandes Figueira/Fiocruz. Rio de Janeiro – RJ – Brasil; ii Instituto Nacional de Saúde da Mulher, da Criança e do Adolescente Fernandes Figueira/Fiocruz. Rio de Janeiro – RJ – Brasil

In the early twentieth century, infant mortality, the abandonment of children by poor families, and the exploitation of child labor were seen as serious problems that were diverting the country from its developmental aspirations. A number of physicians, legislators, and legal experts put great effort into getting the “problems of childhood” included in the political agenda. A key figure amongst these actors was the pediatrician Antônio Fernandes Figueira, whose endeavors resulted in the creation of institutions and public policies for mothers and infants.

An important player in the field of Brazilian pediatrics, Fernandes Figueira advocated for preventive health for children, science education for mothers, and the crucial importance of breastfeeding; essentially, integrated care for mothers and their offspring ( Sanglard, 2014 ). During the public health reform between 1920 and 1923, one result of which was the creation of the Child Health Inspectorate, Fernandes Figueira was put in charge of setting up child health units in several districts of the then Brazilian capital, Rio de Janeiro, as well as the building a new hospital, Hospital Abrigo Arthur Bernardes, between 1924 and 1926.

Opened in April 1926 with much pomp and circumstance and heralded by the press as a new benchmark in healthcare, combining as it did care for mothers and their children, the hospital did not prosper. In 1928, when Fernandes Figueira died, it entered into rapid decline and was shut down in 1935. Only at the end of the 1930s, during the Getúlio Vargas dictatorship and the growing popularity of eugenic beliefs around childhood, was it reopened, and in 1940 it was incorporated into the recently formed National Department for Children, part of the Ministry of Education and Health. As a pillar of this department, the hospital’s mission was expanded to include responsibility for research into problems associated with maternity and child health. In 1946, it was renamed the Fernandes Figueira Institute (Instituto Fernandes Figueira, IFF) ( Ribeiro, 2020 ).

The 1950s was a golden age for IFF. A vibrant pediatric hub, it became a hotbed for some of the country’s most prominent pediatricians. For a long time, it also housed the Brazilian Pediatrics Society. Furthermore, it spawned some truly groundbreaking initiatives, such as the creation of Brazil’s first ever blood bank and first ever human milk bank, as well as being the second institution in the country to offer pediatric residencies.

In 1970, the federal government created the Oswaldo Cruz Foundation (Fundação Instituto Oswaldo Cruz, Fiocruz), which became the umbrella organization for several biomedical institutes, including IFF. As part of the new foundation, it became a designated unit for women’s and children’s health, which resulted in some profound institutional transformations. As part of Fiocruz, it had to invest in research, improve the quality of healthcare, provide training for health workers, and upgrade its teaching activities.

One area that featured among the various actions taken in response to the new demands was neonatal care. Alongside developments in neonatology, progress was also achieved in pediatrics and pediatric surgery. As progress in each field fed into the others, it was not long before the institute became a reference in all three. These advances also ended up strengthening other areas, such as genetics, as well as research in and care for patients with rare diseases.

In the following decade, the institute reformulated one of its areas with the greatest social impact: its human milk bank. Initially envisaged as a center for the processing and distribution of human milk, the bank was transformed into a breastfeeding service, focusing on care for mothers and health education initiatives promoting breastfeeding. This expansion of the remit of the human milk bank made IFF a benchmark and hub of good practices and knowledge about breastfeeding.

In 1988, during the major health reform that resulted in the creation of the public health service (Serviço Único de Saúde, SUS), IFF enrolled its first master’s students. A doctoral program followed in 1995. The graduate education programs, together with the medical residencies, which were also expanding, ended up changing the institute’s profile, providing high-level training for its workforce and promoting advanced research.

In the 2000s, the expansion of the institute’s scope of action and research interests became increasingly guided by the needs of SUS. As of 2003, when the Ministry of Health created the National Policy for Humanization, IFF began to invest in a range of initiatives to humanize healthcare, notably a project designed to enable children with complex chronic health issues to be cared for at home, as well as humanized care for newborns and children with chronic diseases.

In regard to its contributions in tackling epidemiological issues both locally and nationally, IFF was particularly important during the zika epidemic in 2015 and 2016. When the disease first broke out, it was not easy to generate enough scientific evidence to prove the association between zika virus and microcephaly. The causal link was ratified in a study by IFF, which consolidated the notion that infection with the zika virus during pregnancy was associated with fetal malformation. The studies into zika carried out at the institute not only had repercussions in scientific circles, but also had a direct influence on public policies and a huge societal impact, helping mothers and children with neurological problems to fight for their rights.

Today, under the new name of Fernandes Figueira National Institute for Women’s, Children’s, and Adolescents’ Health (Instituto Nacional de Saúde da Mulher, da Criança e do Adolescente Fernandes Figueira), IFF operates as a technical and scientific unit of Fiocruz devoted to healthcare, education, research, and technology development. Two of its most important activities with a nationwide footprint are its coordination of the National Network of Human Milk Banks and the Brazilian Neonatal Research Network. As for its work in training and development, it has residencies involving multiple professional disciplines in a range of medical and nursing areas. It also offers master’s and doctoral degrees in women’s and children’s health and applied research, as well as a professional masters in women’s and children’s health. Its graduate programs provide inputs that enable the ongoing improvement of all its work.

In 2024, IFF celebrates its centenary. Incorporated into Fiocruz more than 50 years ago, its work has been guided by the institution’s mission to produce knowledge and technology for the public health system, SUS. Synergically, IFF has expanded the institutional remit of Fiocruz into the field of women’s, children’s, and adolescents’ health, while its own educational and research activities have been consolidated.

One hundred years since its creation, IFF is keen to reassess what direction it should take to best respond to the challenges posed in the contemporary context. One part of this process involves looking back at its past to help guide how best to move forward. As part of this endeavor to gather historical knowledge and sources on the history of IFF, we present our readers with interviews with two key players who are engaged in reviving the history of the institute in the fields of education and neonatology, as well as two articles on the institute’s history and on groups of professionals who helped shape that history.
